# μ-Oxido-bis­[hydridotris(tri­methyl­phosphane-κ*P*)iridium(III)](*Ir*—*Ir*) bis­(tetra­fluorido­borate) dihydrate

**DOI:** 10.1107/S160053681400453X

**Published:** 2014-03-08

**Authors:** Joseph Merola, Trang Le Husebo

**Affiliations:** a Department of Chemistry, Virginia Tech, Blacksburg, VA 24061, USA

## Abstract

The title compound, [Ir_2_H_2_O(C_3_H_9_P)_6_](BF_4_)_2_·2H_2_O, was isolated from the reaction between [Ir(COD)(PMe_3_)_3_]BF_4_ and H_2_ in water (COD is cyclo­octa-1,5-diene). The asymmetric unit consists of one Ir^III^ atom bonded to three PMe_3_ groups, one hydride ligand and half an oxide ligand, in addition to a BF_4_^−^ counter-ion and one water molecule of hydration. The single oxide ligand bridging two Ir^III^ atoms is disordered across an inversion center with each O atom having a 50% site occupancy. Each Ir^III^ atom has three PMe_3_ groups occupying facial positions, with the half-occupancy O atoms, a hydride ligand and an Ir—Ir bond completing the coordination sphere. The Ir—Ir distance is 2.8614 (12) Å, comparable to other iridium(III) metal–metal bonds. Two water mol­ecules hydrogen bond to two BF_4_^−^ anions in the unit cell.

## Related literature   

For previous work on the aqueous chemistry of Ir(H)_2_(Cl)(PMe_3_)_3_, see: Merola *et al.* (2012 ▶). For the synthesis of [Ir(COD)(PMe_3_)_3_]BF_4_, see: Frazier & Merola (1992 ▶). For an Ir—Ir bond bridged only by an oxide, see: McGhee *et al.* (1988 ▶). For Ir—Ir bonds bridged by hydroxide and methoxide ligands, see: Fujita *et al.* (2000 ▶) (CCDC deposition numbers 146417–146418). For an Ir—Ir bond bridged by a phenoxide group, see: Lee *et al.* (2009 ▶) (CCDC deposition number 729562). For an Ir—Ir bond bridged by an oxide and a phenyl­imido group, see: Dobbs & Bergman (1994 ▶) (CCDC deposition number 645882). For a classic discussion of the *trans* effect and *trans* influence, see: Hartley (1973 ▶). For a description of the Cambridge Crystallographic Database, see: Groom & Allen (2014 ▶).
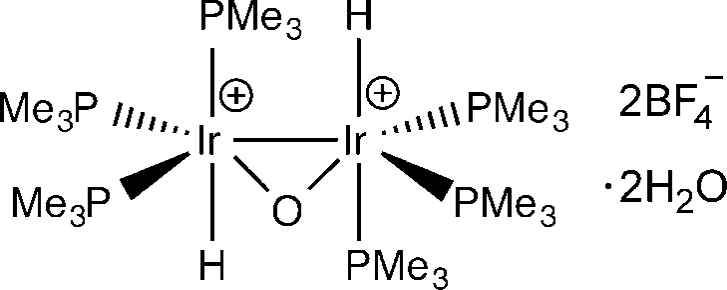


## Experimental   

### 

#### Crystal data  

[Ir_2_H_2_O(C_3_H_9_P)_6_](BF_4_)_2_·2H_2_O
*M* *_r_* = 1068.50
Triclinic, 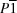

*a* = 9.2686 (19) Å
*b* = 9.6491 (19) Å
*c* = 11.082 (2) Å
α = 96.48 (3)°
β = 97.81 (3)°
γ = 97.97 (3)°
*V* = 963.6 (3) Å^3^
*Z* = 1
Mo *K*α radiation
μ = 7.21 mm^−1^
*T* = 298 K
0.5 × 0.3 × 0.2 mm

#### Data collection  

Siemens P4 diffractometer
Absorption correction: ψ scan (North *et al.*, 1968 ▶) *T*_min_ = 0.293, *T*_max_ = 0.420
4707 measured reflections
4430 independent reflections
3949 reflections with *I* > 2σ(*I*)
*R*_int_ = 0.015
3 standard reflections every 300 reflections intensity decay: 0.0 (1)

#### Refinement  

*R*[*F* ^2^ > 2σ(*F* ^2^)] = 0.034
*wR*(*F* ^2^) = 0.086
*S* = 1.10
4430 reflections
196 parameters
1 restraint
H atoms treated by a mixture of independent and constrained refinement
Δρ_max_ = 1.04 e Å^−3^
Δρ_min_ = −1.45 e Å^−3^

### 

Data collection: *XSCANS* (Siemens, 1996 ▶); cell refinement: *XSCANS*; data reduction: *XSCANS*; program(s) used to solve structure: *SHELXS97* (Sheldrick, 2008 ▶); program(s) used to refine structure: *SHELXL97* (Sheldrick, 2008 ▶); molecular graphics: *OLEX2* (Dolomanov *et al.*, 2009 ▶); software used to prepare material for publication: *OLEX2*.

## Supplementary Material

Crystal structure: contains datablock(s) I. DOI: 10.1107/S160053681400453X/pk2518sup1.cif

Structure factors: contains datablock(s) I. DOI: 10.1107/S160053681400453X/pk2518Isup2.hkl

Supporting information file. DOI: 10.1107/S160053681400453X/pk2518Isup3.mol

CCDC reference: 988922

Additional supporting information:  crystallographic information; 3D view; checkCIF report

## Figures and Tables

**Table 1:** Hydrogen-bond geometry (Å, °)

*D*—H⋯*A*	*D*—H	H⋯*A*	*D*⋯*A*	*D*—H⋯*A*
O2—H2*A*⋯F1^i^	0.85	2.08	2.856 (15)	152
O2—H2*B*⋯F4^ii^	0.85	1.96	2.804 (18)	170

Symmetry codes: (i) 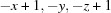; (ii) 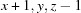.

**Fig. 1.**
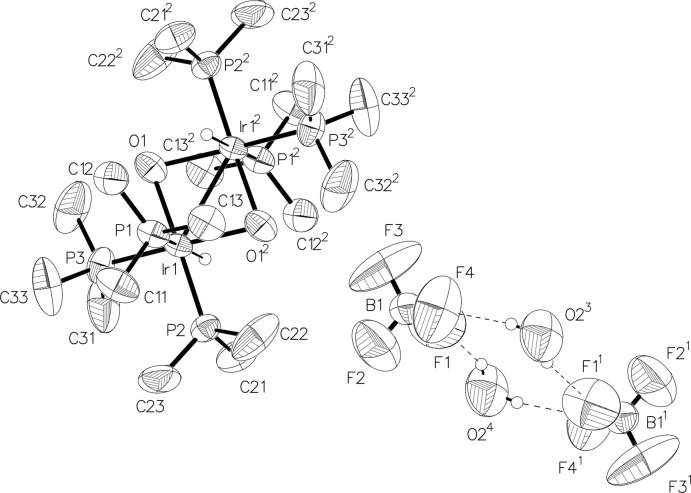
Thermal ellipsoid plot of the structure with ellipsoids drawn at the 50% probability level. Atoms are labeled with superscripts to indicate the symmetry operator that generated them. A1: –X,-Y,2-Z, A2: 1-X,1-Y,1-Z, A3: -1+X,+Y,1+Z, A4: 1-X,-Y,1-Z.

**Fig. 2.**
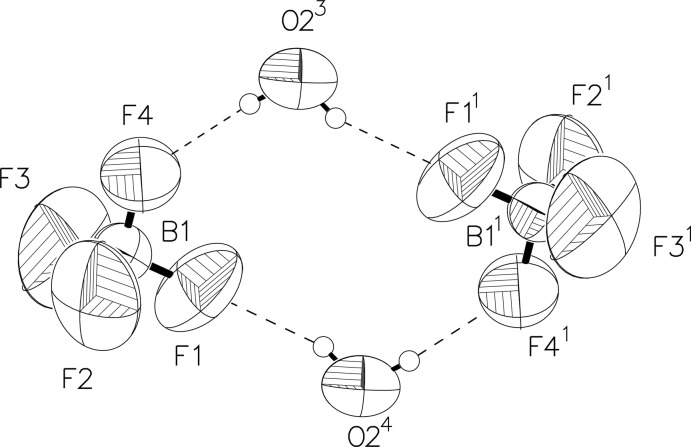
The hydrogen-bonding between water and the tetrafluoroborate anion in the title compound. Ellipsoids are drawn at the 50% probability level and atoms are labeled with supercripts to indicate the symmetry operator that generated them. A1: –X,-Y,2-Z, A2: 1-X,1-Y,1-Z, A3: -1+X,+Y,1+Z, A4: 1-X,-Y,1-Z.

## supplementary crystallographic information

### 1. Comment

Our studies of the aqueous catalysis of iridium trimethylphosphine complexes led us to a detailed study of the solution behavior and catalytic chemistry of H_2_Ir(PMe_3_)_3_Cl in water (Merola *et al.*, 2012). H_2_Ir(PMe_3_)_3_Cl is formed by the reaction between [Ir(COD)(PMe_3_)_3_]Cl and H_2_ in water (COD = 1,5-cyclooctadiene). The reaction of H_2_ with [Ir(COD)(PMe_3_)_3_][BF_4_] (Frazier & Merola, 1992) was investigated to see how a poorly coordinating anion will affect that system. The reaction of hydrogen gas with the BF_4_^-^ salt did not lead to a clean product, but to a yellow solid that, according to its ^1^H NMR spectrum, is a very complex mixture of products. The hydride region of the spectrum was particularly complicated with many hydride resonances from δ -10 to -40 p.p.m.. Slow cooling of an aqueous solution of the reaction mixture produced a few crystals suitable for X-ray diffraction. The title compound is a dinuclear iridium complex with an iridium—iridium bond bridged by a single oxo bridge. Iridium hydride hydrogen atoms were located *via* a difference map and set at a fixed Ir—H distance of 1.55 Å with *U*_iso_ set at 1.5 the *U*_eq_ of iridium. The oxo bridge is disordered about the inversion center. The Ir—P bond distances are 2.2524 (19), 2.258 (2), and 2.4001 (17) Å with the longer bond length for P *trans* to H which has a large *trans* influence (Hartley, 1973). The dinuclear iridium fragment is a dication charge balanced by two BF_4_^-^ ions that show the large thermal ellipsoids common for fluorinated anions. There are two waters of hydration in the crystal lattice that are hydrogen-bonded to the BF_4_^-^ ions.

There were some issues with making correct assignments for the title compound in the absence of clear spectral identification. One must be cautious in the assignment of hydrogen attached to a heavy metal such as iridium, but the presence of iridium hydrides is consistent with one set of resonances found in the ^1^H NMR spectrum at δ -11 p.p.m. as a doublet of triplets due to coupling to the hydrogen from one *trans* P atom (larger doublet coupling) and two *cis* P atoms (smaller triplet coupling). The larger Ir—P bond distance for one of the PMe_3_ ligands is also consistent with a hydride ligand situated *trans* to it. That the bridging ligand is a single oxo bridge may be inferred by the fact that the iridium fragment is a dication - *two* oxo bridges would make the fragment neutral. Attempts were made to solve and refine the structure in P1 to try and resolve the issue of the disordered oxo group, but working in P1 led to a significantly poorer structure model. In addition, models with bridging hydroxides were attempted and also found to be unsatisfactory.

There are 783 structures in the CSD with Ir—Ir bonds, but when limited to those also bridged by oxygen, there are only 6 entries (Groom & Allen, 2014). Two of the structures contained bridging hydroxide and methoxide (Fujita *et al.*, 2000) with Ir—Ir distances of 2.953 (1) and 2.933 (4) Å respectively. An Ir—Ir bond bridged by a phenoxide ligand had an Ir—Ir distance of 2.902 (4) Å (Lee *et al.*, 2009). An Ir—Ir bond with a bridging oxide with no other bridging ligand displayed an Ir—Ir bond of 2.6172 (4) Å (McGhee *et al.*, 1988) and two iridium atoms bridged with both an oxide and a phenylimido ligand showed an Ir—Ir distance of 2.7220 (2) Å (Dobbs & Bergman, 1994). The Ir—Ir distance in the title compound of 2.8614 (12) Å is consistent with a single metal-metal bond bridged by an oxide rather than an alkoxide or hydroxide.

### 2. Experimental

[Ir(COD)(PMe_3_)_3_][BF_4_] (0.100 g) (Frazier & Merola, 1992) was dissolved in 10 ml of degassed, distilled H_2_O and heated to reflux while bubbling H_2_ through the solution. At the end of 2 h reflux, the reaction mixture was a yellow, homogeneous solution. Removal of the solvent under reduced pressure yielded 0.062 g of a yellow powder with complicated NMR spectra indicating the presence of multiple products. Dissolving the yellow powder in water and allowing for the slow evaporation of the solution yielded a few crystals suitable for X-ray diffraction. Screening 3 of the dozen or so crytals all showed the same unit cell.

### 3. Refinement

Trimethylphosphine H atoms were placed at calculated positions and refined using a model in which the H atoms ride on the carbon atom to which it is attached with *U*_iso_(H) = 1.5U_eq_(C). The metal hydride hydrogen atoms were placed based on electron density from a difference map, the distance fixed to 1.55 Å and *Uiso*(H)=1.5Ueq(Ir) but the coordinates were allowed to refine. Hydrogen atoms on water were placed based on hydrogen-bonding vectors, *U*_iso_(H) = 1.5U_eq_(O) and the water was treated as a rigid group in refinement.

### Crystal data

**Table:**

[Ir_2_H_2_O(C_3_H_9_P)_6_](BF_4_)_2_·2H_2_O	*Z* = 1
*M**_r_* = 1068.50	*F*(000) = 518
Triclinic, *P*1	*D*_x_ = 1.841 Mg m^−^^3^
*a* = 9.2686 (19) Å	Mo *K*α radiation, λ = 0.71073 Å
*b* = 9.6491 (19) Å	Cell parameters from 35 reflections
*c* = 11.082 (2) Å	θ = 2–22°
α = 96.48 (3)°	µ = 7.21 mm^−^^1^
β = 97.81 (3)°	*T* = 298 K
γ = 97.97 (3)°	Prism, clear light yellow
*V* = 963.6 (3) Å^3^	0.5 × 0.3 × 0.2 mm

### Data collection

**Table:**

Siemens P4 diffractometer	3949 reflections with *I* > 2σ(*I*)
Radiation source: fine-focus sealed tube, Siemens P4	*R*_int_ = 0.015
Graphite monochromator	θ_max_ = 27.5°, θ_min_ = 1.9°
ω scans	*h* = 0→12
Absorption correction: ψ scan (North *et al.*, 1968)	*k* = −12→12
*T*_min_ = 0.293, *T*_max_ = 0.420	*l* = −14→14
4707 measured reflections	3 standard reflections every 300 reflections
4430 independent reflections	intensity decay: 0.0(1)

### Refinement

**Table:**

Refinement on *F*^2^	Primary atom site location: heavy-atom method
Least-squares matrix: full	Secondary atom site location: difference Fourier map
*R*[*F*^2^ > 2σ(*F*^2^)] = 0.034	Hydrogen site location: inferred from neighbouring sites
*wR*(*F*^2^) = 0.086	H atoms treated by a mixture of independent and constrained refinement
*S* = 1.10	*w* = 1/[σ^2^(*F*_o_^2^) + (0.0449*P*)^2^ + 1.2979*P*] where *P* = (*F*_o_^2^ + 2*F*_c_^2^)/3
4430 reflections	(Δ/σ)_max_ < 0.001
196 parameters	Δρ_max_ = 1.04 e Å^−^^3^
1 restraint	Δρ_min_ = −1.45 e Å^−^^3^

### Special details

**Table:**

Geometry. All e.s.d.'s (except the e.s.d. in the dihedral angle between two l.s. planes) are estimated using the full covariance matrix. The cell e.s.d.'s are taken into account individually in the estimation of e.s.d.'s in distances, angles and torsion angles; correlations between e.s.d.'s in cell parameters are only used when they are defined by crystal symmetry. An approximate (isotropic) treatment of cell e.s.d.'s is used for estimating e.s.d.'s involving l.s. planes.
Refinement. Refinement of *F*^2^ against ALL reflections. The weighted *R*-factor *wR* and goodness of fit *S* are based on *F*^2^, conventional *R*-factors *R* are based on *F*, with *F* set to zero for negative *F*^2^. The threshold expression of *F*^2^ > σ(*F*^2^) is used only for calculating *R*-factors(gt) *etc*. and is not relevant to the choice of reflections for refinement. *R*-factors based on *F*^2^ are statistically about twice as large as those based on *F*, and *R*- factors based on ALL data will be even larger.

### Fractional atomic coordinates and isotropic or equivalent isotropic displacement parameters (Å^2^)

**Table:**

	*x*	*y*	*z*	*U*_iso_*/*U*_eq_	Occ. (<1)
Ir1	0.44635 (2)	0.39247 (2)	0.397713 (19)	0.04330 (8)	
H	0.502 (7)	0.285 (6)	0.477 (5)	0.065*	
P1	0.30869 (19)	0.54619 (17)	0.29012 (15)	0.0545 (4)	
P2	0.26109 (19)	0.20850 (19)	0.36625 (17)	0.0573 (4)	
P3	0.5672 (2)	0.3085 (2)	0.24848 (19)	0.0650 (5)	
O1	0.6514 (12)	0.5458 (12)	0.4336 (9)	0.071 (3)	0.50
C11	0.1914 (11)	0.4787 (10)	0.1441 (8)	0.091 (3)	
H11A	0.2482	0.4349	0.0888	0.137*	
H11B	0.1530	0.5552	0.1092	0.137*	
H11C	0.1113	0.4104	0.1572	0.137*	
C12	0.4183 (10)	0.6996 (8)	0.2485 (7)	0.077 (2)	
H12A	0.4619	0.7628	0.3215	0.115*	
H12B	0.3563	0.7471	0.1960	0.115*	
H12C	0.4947	0.6699	0.2059	0.115*	
C13	0.1780 (9)	0.6263 (9)	0.3741 (8)	0.076 (2)	
H13A	0.1068	0.5534	0.3938	0.114*	
H13B	0.1284	0.6857	0.3241	0.114*	
H13C	0.2300	0.6819	0.4486	0.114*	
C21	0.2986 (10)	0.0608 (9)	0.4459 (10)	0.089 (3)	
H21A	0.3734	0.0170	0.4114	0.133*	
H21B	0.2101	−0.0068	0.4370	0.133*	
H21C	0.3321	0.0936	0.5315	0.133*	
C22	0.0959 (11)	0.2463 (12)	0.4220 (15)	0.133 (5)	
H22A	0.1169	0.2749	0.5092	0.200*	
H22B	0.0224	0.1633	0.4043	0.200*	
H22C	0.0600	0.3210	0.3823	0.200*	
C23	0.1925 (15)	0.1187 (14)	0.2126 (10)	0.153 (6)	
H23A	0.1346	0.1767	0.1678	0.230*	
H23B	0.1325	0.0306	0.2172	0.230*	
H23C	0.2741	0.1012	0.1713	0.230*	
C31	0.6143 (17)	0.1367 (12)	0.2590 (11)	0.136 (6)	
H31A	0.6139	0.1158	0.3416	0.203*	
H31B	0.7107	0.1338	0.2372	0.203*	
H31C	0.5435	0.0681	0.2038	0.203*	
C32	0.7546 (11)	0.4063 (16)	0.2693 (13)	0.139 (5)	
H32A	0.7517	0.5056	0.2861	0.208*	
H32B	0.7968	0.3878	0.1958	0.208*	
H32C	0.8137	0.3769	0.3369	0.208*	
C33	0.5107 (14)	0.3194 (16)	0.0923 (9)	0.129 (5)	
H33A	0.4247	0.2506	0.0615	0.194*	
H33B	0.5886	0.3015	0.0466	0.194*	
H33C	0.4881	0.4122	0.0834	0.194*	
F1	0.1765 (12)	0.0878 (10)	0.8932 (9)	0.183 (4)	
F2	0.0995 (18)	0.1122 (13)	0.7112 (9)	0.239 (6)	
F3	0.2533 (12)	0.2732 (13)	0.8167 (16)	0.276 (8)	
F4	0.0302 (11)	0.2307 (12)	0.8445 (11)	0.195 (5)	
B1	0.1475 (12)	0.1797 (12)	0.8151 (11)	0.082 (3)	
O2	0.8397 (15)	0.1785 (12)	0.0159 (12)	0.152 (4)	
H2A	0.8300	0.0897	0.0167	0.227*	
H2B	0.9030	0.2028	−0.0295	0.227*	

### Atomic displacement parameters (Å^2^)

**Table:**

	*U*^11^	*U*^22^	*U*^33^	*U*^12^	*U*^13^	*U*^23^
Ir1	0.04201 (12)	0.03886 (12)	0.04581 (13)	0.01001 (8)	−0.00422 (8)	0.00061 (8)
P1	0.0565 (9)	0.0488 (8)	0.0551 (9)	0.0168 (7)	−0.0087 (7)	0.0040 (7)
P2	0.0473 (8)	0.0595 (9)	0.0631 (10)	0.0015 (7)	0.0047 (7)	0.0133 (8)
P3	0.0593 (10)	0.0768 (12)	0.0724 (11)	0.0272 (9)	0.0252 (9)	0.0294 (9)
O1	0.073 (6)	0.078 (7)	0.054 (5)	0.013 (5)	−0.004 (5)	−0.001 (5)
C11	0.091 (6)	0.092 (6)	0.080 (5)	0.032 (5)	−0.034 (5)	−0.003 (5)
C12	0.087 (6)	0.072 (5)	0.069 (5)	0.014 (4)	−0.005 (4)	0.019 (4)
C13	0.061 (4)	0.081 (5)	0.088 (5)	0.033 (4)	0.000 (4)	0.008 (4)
C21	0.083 (6)	0.062 (5)	0.122 (8)	0.005 (4)	0.009 (5)	0.031 (5)
C22	0.069 (6)	0.111 (8)	0.252 (17)	0.032 (6)	0.075 (8)	0.078 (10)
C23	0.159 (12)	0.161 (11)	0.088 (7)	−0.101 (10)	−0.029 (7)	0.012 (7)
C31	0.215 (15)	0.115 (8)	0.129 (9)	0.105 (10)	0.103 (10)	0.044 (7)
C32	0.065 (6)	0.221 (16)	0.147 (11)	0.021 (8)	0.041 (7)	0.066 (11)
C33	0.139 (10)	0.215 (14)	0.064 (5)	0.094 (10)	0.036 (6)	0.040 (7)
F1	0.223 (10)	0.145 (7)	0.182 (8)	0.062 (7)	−0.027 (7)	0.050 (6)
F2	0.349 (18)	0.228 (12)	0.105 (6)	−0.025 (12)	0.014 (8)	−0.015 (7)
F3	0.136 (8)	0.222 (12)	0.46 (2)	−0.071 (8)	0.055 (11)	0.102 (14)
F4	0.173 (8)	0.227 (11)	0.245 (11)	0.118 (8)	0.097 (8)	0.110 (9)
B1	0.077 (6)	0.080 (6)	0.086 (7)	0.003 (5)	0.013 (5)	0.003 (5)
O2	0.179 (10)	0.139 (8)	0.165 (9)	0.073 (8)	0.071 (7)	0.027 (7)

### Geometric parameters (Å, º)

**Table:**

Ir1—Ir1^i^	2.8614 (12)	C13—H13C	0.9600
Ir1—H	1.53 (2)	C21—H21A	0.9600
Ir1—P1	2.4000 (17)	C21—H21B	0.9600
Ir1—P2	2.2524 (19)	C21—H21C	0.9600
Ir1—P3	2.258 (2)	C22—H22A	0.9600
Ir1—O1^i^	2.242 (10)	C22—H22B	0.9600
Ir1—O1	2.200 (11)	C22—H22C	0.9600
P1—C11	1.820 (8)	C23—H23A	0.9600
P1—C12	1.817 (8)	C23—H23B	0.9600
P1—C13	1.827 (8)	C23—H23C	0.9600
P2—C21	1.808 (8)	C31—H31A	0.9600
P2—C22	1.794 (9)	C31—H31B	0.9600
P2—C23	1.809 (11)	C31—H31C	0.9600
P3—C31	1.784 (9)	C32—H32A	0.9600
P3—C32	1.830 (11)	C32—H32B	0.9600
P3—C33	1.759 (10)	C32—H32C	0.9600
O1—Ir1^i^	2.242 (10)	C33—H33A	0.9600
C11—H11A	0.9600	C33—H33B	0.9600
C11—H11B	0.9600	C33—H33C	0.9600
C11—H11C	0.9600	F1—B1	1.336 (14)
C12—H12A	0.9600	F2—B1	1.249 (14)
C12—H12B	0.9600	F3—B1	1.234 (13)
C12—H12C	0.9600	F4—B1	1.318 (13)
C13—H13A	0.9600	O2—H2A	0.8500
C13—H13B	0.9600	O2—H2B	0.8499
			
Ir1^i^—Ir1—H	88 (3)	H12B—C12—H12C	109.5
P1—Ir1—Ir1^i^	92.32 (5)	P1—C13—H13A	109.5
P1—Ir1—H	167 (3)	P1—C13—H13B	109.5
P2—Ir1—Ir1^i^	132.22 (5)	P1—C13—H13C	109.5
P2—Ir1—H	75 (3)	H13A—C13—H13B	109.5
P2—Ir1—P1	95.38 (7)	H13A—C13—H13C	109.5
P2—Ir1—P3	95.89 (8)	H13B—C13—H13C	109.5
P3—Ir1—Ir1^i^	128.86 (6)	P2—C21—H21A	109.5
P3—Ir1—H	89 (3)	P2—C21—H21B	109.5
P3—Ir1—P1	100.31 (7)	P2—C21—H21C	109.5
O1—Ir1—Ir1^i^	50.6 (3)	H21A—C21—H21B	109.5
O1^i^—Ir1—Ir1^i^	49.2 (3)	H21A—C21—H21C	109.5
O1^i^—Ir1—H	81 (3)	H21B—C21—H21C	109.5
O1—Ir1—H	97 (3)	P2—C22—H22A	109.5
O1—Ir1—P1	93.5 (3)	P2—C22—H22B	109.5
O1^i^—Ir1—P1	89.5 (3)	P2—C22—H22C	109.5
O1—Ir1—P2	170.5 (3)	H22A—C22—H22B	109.5
O1^i^—Ir1—P2	83.7 (3)	H22A—C22—H22C	109.5
O1^i^—Ir1—P3	170.2 (3)	H22B—C22—H22C	109.5
O1—Ir1—P3	79.2 (3)	P2—C23—H23A	109.5
O1—Ir1—O1^i^	99.8 (4)	P2—C23—H23B	109.5
C11—P1—Ir1	120.0 (3)	P2—C23—H23C	109.5
C11—P1—C13	100.5 (4)	H23A—C23—H23B	109.5
C12—P1—Ir1	115.3 (3)	H23A—C23—H23C	109.5
C12—P1—C11	100.7 (4)	H23B—C23—H23C	109.5
C12—P1—C13	102.1 (4)	P3—C31—H31A	109.5
C13—P1—Ir1	115.4 (3)	P3—C31—H31B	109.5
C21—P2—Ir1	114.9 (3)	P3—C31—H31C	109.5
C21—P2—C23	100.6 (6)	H31A—C31—H31B	109.5
C22—P2—Ir1	114.6 (4)	H31A—C31—H31C	109.5
C22—P2—C21	100.8 (5)	H31B—C31—H31C	109.5
C22—P2—C23	102.8 (7)	P3—C32—H32A	109.5
C23—P2—Ir1	120.3 (4)	P3—C32—H32B	109.5
C31—P3—Ir1	115.4 (3)	P3—C32—H32C	109.5
C31—P3—C32	97.9 (7)	H32A—C32—H32B	109.5
C32—P3—Ir1	109.8 (5)	H32A—C32—H32C	109.5
C33—P3—Ir1	122.3 (4)	H32B—C32—H32C	109.5
C33—P3—C31	107.5 (6)	P3—C33—H33A	109.5
C33—P3—C32	100.0 (6)	P3—C33—H33B	109.5
Ir1—O1—Ir1^i^	80.2 (4)	P3—C33—H33C	109.5
P1—C11—H11A	109.5	H33A—C33—H33B	109.5
P1—C11—H11B	109.5	H33A—C33—H33C	109.5
P1—C11—H11C	109.5	H33B—C33—H33C	109.5
H11A—C11—H11B	109.5	F2—B1—F1	108.4 (11)
H11A—C11—H11C	109.5	F2—B1—F4	102.0 (12)
H11B—C11—H11C	109.5	F3—B1—F1	112.7 (12)
P1—C12—H12A	109.5	F3—B1—F2	114.0 (13)
P1—C12—H12B	109.5	F3—B1—F4	112.5 (12)
P1—C12—H12C	109.5	F4—B1—F1	106.4 (10)
H12A—C12—H12B	109.5	H2A—O2—H2B	109.5
H12A—C12—H12C	109.5		
			
Ir1^i^—Ir1—P1—C11	−176.0 (4)	P2—Ir1—P3—C33	78.6 (6)
Ir1^i^—Ir1—P1—C12	63.3 (3)	P3—Ir1—P1—C11	53.8 (4)
Ir1^i^—Ir1—P1—C13	−55.5 (3)	P3—Ir1—P1—C12	−66.9 (3)
Ir1^i^—Ir1—P2—C21	−75.3 (4)	P3—Ir1—P1—C13	174.3 (3)
Ir1^i^—Ir1—P2—C22	40.9 (5)	P3—Ir1—P2—C21	85.6 (4)
Ir1^i^—Ir1—P2—C23	164.2 (7)	P3—Ir1—P2—C22	−158.2 (5)
Ir1^i^—Ir1—P3—C31	106.5 (6)	P3—Ir1—P2—C23	−34.8 (7)
Ir1^i^—Ir1—P3—C32	−2.9 (5)	P3—Ir1—O1—Ir1^i^	−170.1 (3)
Ir1^i^—Ir1—P3—C33	−119.5 (6)	O1—Ir1—P1—C11	133.4 (5)
P1—Ir1—P2—C21	−173.4 (4)	O1^i^—Ir1—P1—C11	−126.8 (5)
P1—Ir1—P2—C22	−57.2 (5)	O1^i^—Ir1—P1—C12	112.5 (4)
P1—Ir1—P2—C23	66.2 (7)	O1—Ir1—P1—C12	12.7 (4)
P1—Ir1—P3—C31	−152.0 (6)	O1^i^—Ir1—P1—C13	−6.3 (4)
P1—Ir1—P3—C32	98.6 (5)	O1—Ir1—P1—C13	−106.1 (4)
P1—Ir1—P3—C33	−18.0 (6)	O1^i^—Ir1—P2—C21	−84.5 (5)
P1—Ir1—O1—Ir1^i^	90.1 (2)	O1^i^—Ir1—P2—C22	31.7 (6)
P2—Ir1—P1—C11	−43.2 (4)	O1^i^—Ir1—P2—C23	155.1 (7)
P2—Ir1—P1—C12	−163.9 (3)	O1—Ir1—P3—C31	116.3 (7)
P2—Ir1—P1—C13	77.3 (3)	O1—Ir1—P3—C32	6.9 (5)
P2—Ir1—P3—C31	−55.4 (6)	O1—Ir1—P3—C33	−109.6 (7)
P2—Ir1—P3—C32	−164.9 (5)	O1^i^—Ir1—O1—Ir1^i^	0.0

Symmetry code: (i) −*x*+1, −*y*+1, −*z*+1.

### Hydrogen-bond geometry (Å, º)

**Table:**

*D*—H···*A*	*D*—H	H···*A*	*D*···*A*	*D*—H···*A*
O2—H2*A*···F1^ii^	0.85	2.08	2.856 (15)	152
O2—H2*B*···F4^iii^	0.85	1.96	2.804 (18)	170

Symmetry codes: (ii) −*x*+1, −*y*, −*z*+1; (iii) *x*+1, *y*, *z*−1.
